# In Vitro & In Vivo Studies on Identifying and Designing Temporin-1CEh from the Skin Secretion of *Rana chensinensis* as the Optimised Antibacterial Prototype Drug

**DOI:** 10.3390/pharmaceutics14030604

**Published:** 2022-03-10

**Authors:** Zhuming Ye, Xiaowei Zhou, Xinping Xi, Yu Zai, Mei Zhou, Xiaoling Chen, Chengbang Ma, Tianbao Chen, Lei Wang, Hang Fai Kwok

**Affiliations:** 1 Institute of Translational Medicine, Faculty of Health Sciences, University of Macau, Taipa, Macau SAR 999078, China; zye04@qub.ac.uk (Z.Y.); xzhou06@qub.ac.uk (X.Z.); x.xi@qub.ac.uk (X.X.); yzai01@qub.ac.uk (Y.Z.); 2 Department of Nutrition, Henry Fok School of Food Science and Engineering, Shaoguan University, Shaoguan 512005, China; 3 School of Pharmacy, Queen’s University Belfast, 97 Lisburn Road, Belfast BT9 7BL, UK; m.zhou@qub.ac.uk (M.Z.); x.chen@qub.ac.uk (X.C.); t.chen@qub.ac.uk (T.C.); l.wang@qub.ac.uk (L.W.); 4 Jiangsu Key Laboratory of Biofunctional Molecule, College of Life Sciences and Chemistry, Jiangsu Second Normal University, Nanjing 210013, China

**Keywords:** antimicrobial activity, temporin, branched peptide, *Galleria mellonella* larva model

## Abstract

Amphibian skin secretion is an ideal source of antimicrobial peptides that are difficult to induce drug resistance to due to their membrane-targeting mechanism as a new treatment scheme. In this study, a natural antimicrobial peptide Temporin-1CEh was identified by molecular cloning and mass spectrometry from the skin secretions of the Chinese forest frog (*Rana chensinensis*). Through the study of the structure and biological activity, it was found that Temporin-1CEh was a helical peptide from the Temporin family, and possessed good anti-Gram-positive bacteria activity through the mechanism of membrane destruction. Seven analogues were further designed to obtain broad-spectrum antimicrobial activity and higher stability in different physiological conditions. The results showed that T1CEh-KKPWW showed potent antibacterial activity with significantly increasing the activity against Gram-negative bacteria in vitro and in vivo with low haemolysis. In addition, T1CEh-KKPWW2 showed high sensitivity to the pH, serum or salts conditions, which applied a branched structure to allow the active units of the peptide to accumulate. Even though the haemolytic activity was increased, the stable antibacterial activity made this novel analogue meet the conditions to become a potential candidate in future antimicrobial and antibiofilm applications.

## 1. Introduction

Nowadays, the occurrence of multiple drug resistance of pathogens is increasing, and even some pathogens can evade the treatment of all clinically used antibiotics [1]. The severe situation of multidrug drug resistance makes it urgent to develop new antibiotics [2,3]. After decades of exploration and research on the natural peptide, more and more attention has been paid to their antimicrobial potential. They have a broad spectrum of antimicrobial activity, including gram-positive and gram-negative bacteria, fungi and viruses [4]. Despite the differences in sequence and secondary structure, they usually have less than 50 amino acid residues in length and carry positive charges [5]. At the same time, some peptides share an amphiphilic character with a hydrophilic surface and a hydrophobic surface [6]. The bacteria and fungi develop resistance to antibiotics through the reduction of drug permeability to their biomembranes, an efflux of antibiotics molecules from the cell, modification of antimicrobial targets, or enzymatic hydrolysis and degradation [7,8,9,10,11,12]. However, an antimicrobial peptide (AMP) inhibits the growth and infection of pathogens through physical destruction or osmosis of the bacterial cell membranes [7]. Such a special antibacterial mechanism makes an AMP and its analogues become candidates to replace the use of traditional antibiotics.

Even so, few AMPs have been approved for clinical use due to considerable drawbacks on specific pathogens, significant haemolysis, high production costs, susceptibility to protease degradation, and decreased activity in the presence of physiological concentrations [8,9]. A balance should be found between hydrophobicity, charge number, helix structure, amphiphilicity, side chain groups and other important factors of AMPs to maximise antibacterial activity and minimise haemolysis [9,10,11]. A number of strategies exist today to deal with the problem of susceptibility, such as the introduction of a ring or branched structure to promote steric hindrance and inhibit protease hydrolysis, or the use of the substitution of certain sites with D-type amino acids or unnatural amino acids [12]. Another strategy is the nanoencapsulation of AMPs [13]. Based on the study of a large number of structure–activity relationships of AMPs, modifying the natural peptide sequences to make them overcome the existing obstacles has become an effective method. The Chinese Forest Frog, *Rana chensinensis,* was used as the research object. Its dried skin, possessing anti-inflammation activity, as well as oviductus ranae are traditional Chinese medicines [14,15]. In addition, it has been found that the natural proteins and peptides extracted from the skin secretions of *Rana chensinensis* have antibacterial, anticancer, antioxidant and anti-apoptotic activities [16,17,18,19]. In this study, the skin secretion of a natural peptide named Temporin-1CEh was identified from the skin secretion of the Chinese Forest Frog by using a combination of ‘shot-gun’ cloning and LC-MS/MS. Several modified strategies including branched structure were used to optimise their antibacterial activity. All chemical synthesised peptides using solid-phase peptide synthesis (SPPS) were applied in the secondary structure and bioactivity, including antibacterial activity in different conditions against ESKAPE pathogens, anti-biofilm activity, permeability and haemolysis to horse red blood cells, as well as in vivo study.

## 2. Materials and Methods

### 2.1. Acquisition of Skin Secretion

Three adult *Rana chensinensis* frogs were obtained from commercial sources and the skin secretion obtaining process was described previously [20]. The study was performed according to the guidelines in the UK Animal (Scientific Procedures) Act 1986, project license PPL 2694, issued by the Department of Health, Social Services and Public Safety, Northern Ireland. Procedures had been vetted by the IACUC of Queen’s University Belfast and approved on 1 March 2011.

### 2.2. Shot-Gun Cloning of Biosynthetic Precursor

The shot-gun cloning was performed to isolate the prepropeptide encoding RNA, as described before [21]. In the 3′-RACE reaction, a nested universal primer (NUP) and a degenerate primer specially designed according to the highly conserved 5′-untranslated region of previously characterised peptide precursor cDNAs from closely-related *Rana* species (5′-ACTTTCYGAWTTRYAAGMCCAAABATG-3′, Y = C + T, W = A + T, R = A + G, M = A + C, B = T + C + G) were used in a segment of the 5′-untranslated region. The degenerate primer was designed relating to the 5′-untranslated high conserved region of *Rana*-species.

### 2.3. Solid-Phase Peptide Synthesis

Temporin-1CEh and seven analogues were synthesised by using a Tribute automated solid-phase peptide synthesiser (Protein Technologies, Tucson, AZ, USA), as described previously [22]. Fmoc-Lys(Fmoc)-OH resin was used in the synthesis of T1CEh-KKPWW2. The concentration of this resin was the same as the forward amino acid resin, and the concentration of the later resins should be twice. The amino groups on the main chain and side chain of lysine are protected by the Fmoc group, and the two peptide chains extend here during the synthesis process. The purification of these peptides was achieved by using the Jupiter C18 semi-prep column and Jupiter C18 column (250 nm × 21.2 mm, Phenomenex, UK) on a Cecil Adept CE4200 HPLC system (Cecil, Cambridge, UK). MALDI-TOF mass spectrometry was used for peptide or HPLC fragment mass analysis using a linear time-of-flight mass spectrometer (Voyager DE, PerSeptive Biosystems, Framingham, MA, USA) in positive detection mode.

### 2.4. Liposomes Preparation

Appropriate amounts of dioleoylphosphatidylcholine (DOPC), dioleoylphosphatidylethanolamine (DOPE) and dioleoylphosphatidylglycerol (DOPG) (Avanti, Tonawanda, NY, USA) were dissolved in chloroform to obtain stock solutions. The desired compositions were mixed and then dried in a high vacuum evaporator until the lipid layer was formed at the bottom of the bottle. The lipid residues were subsequently hydrated at 50 °C with H_2_O. The resulting dispersions were extruded through a stack of two polycarbonate filters (50-nm pore size; Millipore Corp., Bedford, MA, USA) using a Liposofast low pressure homogeniser (Avestin, Ottawa, ON, Canada) to obtain large unilamellar vesicles after five frozen-thaw cycles at 37 °C were performed. The concentration of total phosphorus was determined by the ascorbic acid/ammonium molybdate method, and 3 mM of liposomes was subjected to CD analysis [23,24].

### 2.5. Secondary Structure Analysis

The secondary structure of the peptides was examined by circular dichroism spectropolarimeter (JASCO Inc., Easton, MD, USA) and a quartz cuvette with a 1-mm path length following the previously described parameters setting [25]. Each sample was analysed at 20 °C with the following parameters: scan range of 190–250 nm, scanning speed of 100 nm/min, 1 nm bandwidth, 0.5 nm data pitch and obtained using three accumulations. Peptide samples were dissolved in different environments, including liposomes, SDS/H_2_O (1/99, *w*/*v*) (simulated comparably negatively charged environment of prokaryotic membrane), TFE/H_2_O (50/50, *v*/*v*) (simulated hydrophobic environment of microbial membrane) and H_2_O, respectively.

### 2.6. Antimicrobial Susceptibility Assay

ESKAPE pathogens, encompassing six pathogens which exhibit multidrug resistance and lead nosocomial infections, were used to determine the antibacterial activity of Temporin-1CEh and its analogues by using broth dilution method [26], including Gram-positive bacteria, *S. aureus* (ATCC CRM 6538), MRSA (NCTC 12493) and *E. faecalis* (NCTC 12697), Gram-negative bacteria, *E. coli* (NCTC 10418), *P. aeruginosa* (ATCC 27853) and *K. pneumoniae* (ATCC 43816). A measure of 5 × 10^5^ CFU/mL of bacterial suspension including 2-fold dilution peptides (ranging from 1 µM to 512 µM) was incubated in 96-well plates for 16–20 h at 37 °C. For the positive and negative control, 200 mg/L of final concentration of norfloxacin and 1% DMSO were applied, respectively. The optical density of each well was determined at 550 nm using a Synergy HT plate reader (Biotech, Minneapolis, MN, USA) and the lowest concentration of peptide that resulted in no growth of the bacteria was defined as the MICs. Subsequently, 10 µL of the solution in each clear well was dropped on a Mueller–Hinton agar (MHA) plate and incubated at 37 °C for 16–20 h. The lowest concentration without colony growth was considered as the MBCs.

### 2.7. Biofilm Susceptibility Assay

The anti-biofilm activities were tested on *S. aureus* and *E. coli*, as indicated previously [25]. For the positive and negative control, 20 mg/mL of vancomycin and 1% DMSO were applied, respectively, in both assays. In addition, 0.1% Crystal Violet was used as the biofilm colouring agent.

### 2.8. Membrane Permeability Assay

In order to test the permeability percentage of Temporin-1CEh and its analogues against *S. aureus* and *E. coli*, the SYTOX-GREEN nucleic acid stain was used in the assay indicated before [11]. Tryptic soy broth (TSB) and Luria–Bertani (LB) broth were used as the media for *S. aureus* and *E. coli*, respectively.

### 2.9. Assessing the Impact of Different Environments on Antimicrobial Activity

Mueller–Hinton broth (MHB) at pH 6.0 and 8.0 as well as MHB containing 10% of Fetal Bovine Serum (FBS), 150 mM of NaCl, 1 mM of MgCl_2_ and 4 µM of FeCl_3_, respectively, were prepared. In particular, the pathogens were diluted by the preprepared mediums to a final concentration of 5 × 10^5^ CFU/mL after growing to the logarithmic phase, and other operations followed the MIC assay.

### 2.10. Haemolysis Assay

The erythrocytes from defibrinated horse blood (TCS Biosciences Ltd., Buckingham, UK) were used to perform the haemolysis assay, as described previously [27]. Equal volumes of PBS and 2% (*v*/*v*) of the non-ionic detergent, Triton X-100 (Sigma–Aldrich, St. Louis, MO, USA) in PBS solution, were used as negative and positive controls, respectively. HC_50_ was defined as the mean peptide concentration producing 50% haemolysis.

### 2.11. Assessing the Efficacy of the Peptide Temporin-1CEh, T1CEh-KKPW, T1CEh-KKPWW and T1CEh-KKPWW2 against S. aureus and E. coli In Vivo

In this assay, the larvae of the wax moth (*Galleria mellonella*) were used as the infection model. The larvae (Livefood UK Ltd., Rooks Bridge, UK) were selected between 225 and 275 mg (9 larvae per group in a Petri dish). For the positive and negative controls, 50 mg/kg of vancomycin (25 mg/kg of ampicillin for *E. coli*) and PBS were employed. Final concentration of *S. aureus* and *E. coli* cells injected was 5 × 10^5^ and 5 × 10^6^ CFU/larva, respectively. The assay was performed as described before [28] and the number of alive larvae was recorded every 12 h.

### 2.12. Statistical Analysis

Data were subjected to statistical analysis using Prism (Version 6.0; GraphPad Software Inc., San Diego, CA, USA). Error bars in the graphs represent the standard error of the mean (SEM) with experiments performed on more than three sets of replicates. The significance was determined using Mantel–Cox test for survival curve of wax moth larva, indicated by ns (none significant difference), * (*p* < 0.05), ** (*p* < 0.01) and *** (*p* < 0.001).

## 3. Results

### 3.1. Molecular Cloning of a Novel Peptide Precursor-Encoding cDNA

**Figure 1:**
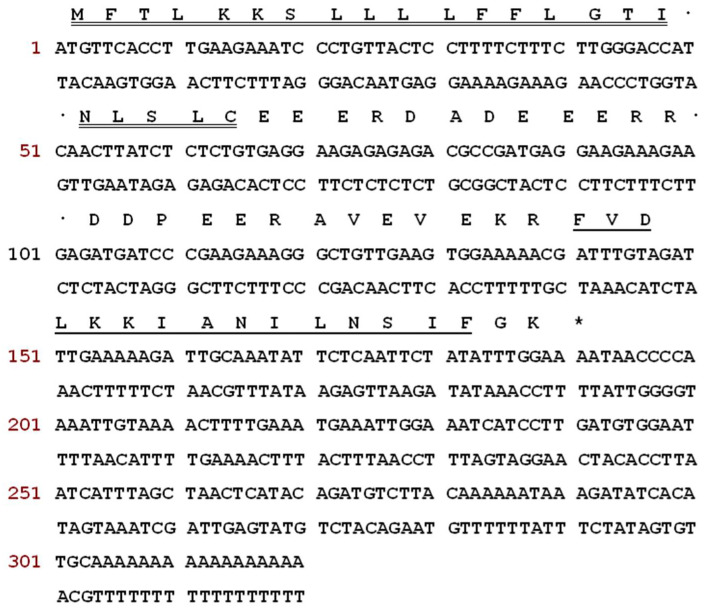
Nucleotide and ORF amino acid sequences of the precursor cDNA which was cloned from the skin secretion library of *Rana chensinensis*. Putative signal peptide sequence is double-underlined, mature peptide sequence is single-underlined and an asterisk represents the stop codon.

**Figure 2:**
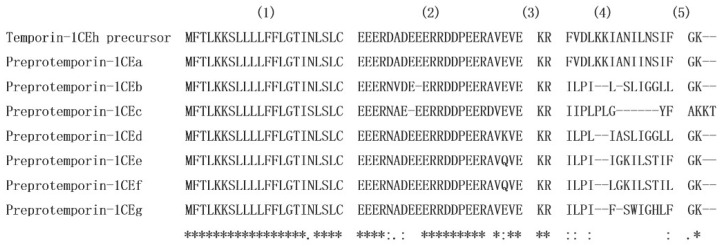
Alignment of open-reading frame amino acid sequences of Temporin-1CEh precursor, preproTemporin-1CEa, preproTemporin-1CEb, preproTemporin-1CEc, preproTemporin-1CEd, preproTemporin-1CEe and preproTemporin-1CEf. Conserved residues are indicated with asterisks. (1) Signal peptide; (2) “spacer” peptide; (3) dibasic residue pro-peptide convertase cleavage site; (4) mature peptide; (5) amide donor for C-terminal amidation of the mature peptide.

The sequences of nucleotide and translated open-reading frame (ORF) amino acids of the precursor cDNA encoding the peptide Temporin-1CEh from the skin secretion library of *Rana chensinensis* is shown in Figure 1. The peptide precursor of Temporin-1CEh contains 64 amino acid residues. Through the bioinformatic alignment using the NCBI-BLAST programme, the precursor peptide shows a high degree of similarity to preproTemporin-1CEa and other six Temporin family prepropeptides from Rana frogs (Figure 2). The topological structure of the precursor can be divided into five putative domains: a highly conserved signal peptide region including 22 amino acid residues, a “spacer” region with rich acidic amino acid residue, a characteristic—Lys-Arg—propeptide convertase cleavage site, a mature peptide region and a C-terminal Gly which acts as an amide donor for C-terminal amidation of mature peptides. For the precursors of Temporin-1CEh and another six analogues, the number of amino acid residues of the ‘mature peptide’ region range from 12 to 15.

### 3.2. Identification of the Peptide from Rana chensinensis Skin Secretion

**Figure 3:**
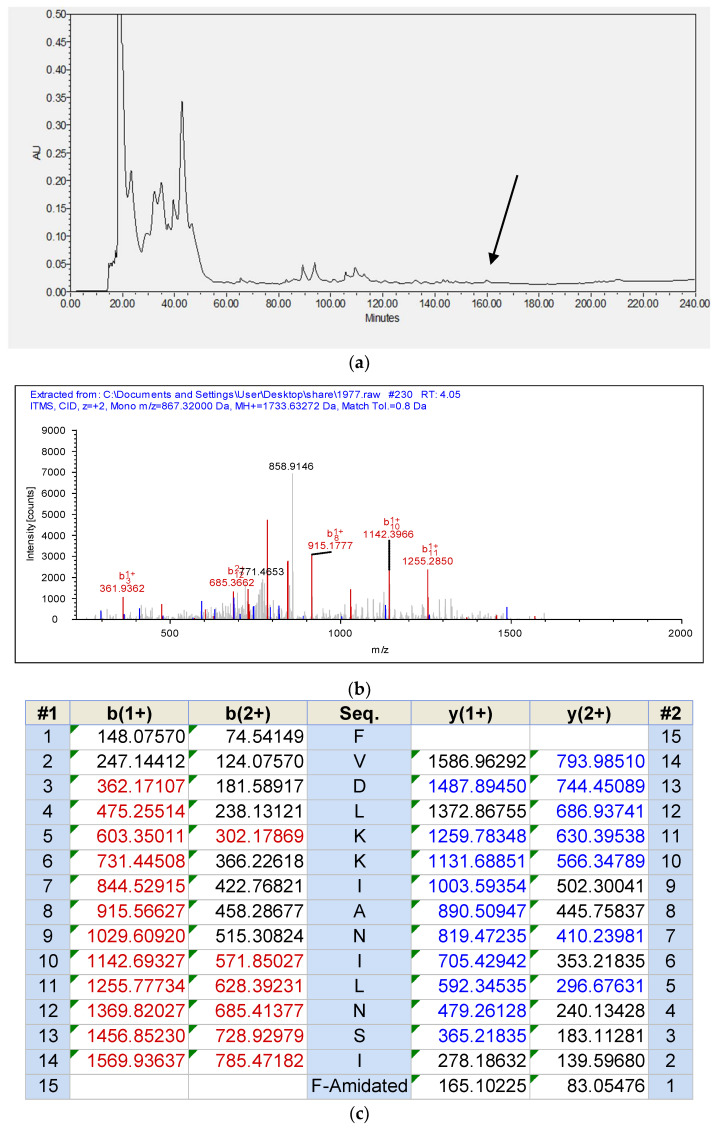
(**a**) RP-HPLC chromatogram of skin secretion of Rana chensinensis. The arrow indicates the retention time of Temporin-1CEh. (**b**) Annotated MS/MS spectrum of Temporin-1CEh. (**c**) Predicted singly- and doubly-charged b-ions and y-ions arising from MS/MS fragmentation of Temporin-1CEh. The observed b- and y-ions are indicated in red and blue typefaces.

With the use of RP-HPLC and mass spectrometry followed later by analysis using MS/MS fragmentation sequencing, Temporin-1CEh was identified in the skin secretion of *Rana chensinensis*. The RP-HPLC chromatography of the skin secretions of *Rana chensinensis*, annotated MS/MS spectrum of Temporin-1CEh and MS/MS fragmentation sequencing analysis, are shown in Figure 3.

### 3.3. Peptide Design following the Tests of Antimicrobial and Haemolytic Activity

**Figure 4:**
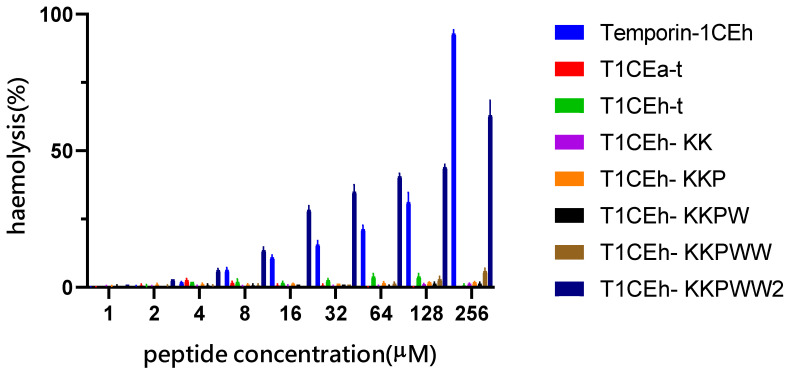
The haemolytic activity of Temporin-1CEh and its analogues at gradient changed concentrations. Positive control and negative control are 1% Triton X-100 and PBS, respectively. Haemolysis rates of positive control and negative controls were 100% and 0%, respectively. Error bars represent the SEM of three replicates.

All sequences of peptides are shown in Table 1. Temporin-1CEh exhibited broad-spectrum antimicrobial activity against all tested ESKAPE strains, whilst it demonstrated a more potent effect against Gram-positive bacteria than Gram-negative bacteria (Table 2). However, the haemolysis rate of Temporin-1CEh reached 92.3% at the concentration of 256 μM (Figure 4). The calculated HC_50_ of Temporin-1CEh was 152.6 μM compared to that of Temporin-1CEa which was 160 μM [29]. Several modified strategies were used to optimise their antibacterial activity.

Firstly, decreasing the haemolytic activity of Temporin-1CEh was the first goal. Four amino acids including isoleucine and phenylalanine at the C-terminus of Temporin-1CEh and Temporin-1CEa were removed in order to decrease the hydrophobicity obtaining T1CEh-t and T1CEa-t. This similar operation in a previous study had ensured the helical structure integrity of the central area [30]. Isoleucine and leucine, hydrophobic amino acids, were retained in the C-terminus of both peptides in order to maintain the hydrophobicity in this region to retain the ability of membrane interaction. In the test of their antimicrobial and haemolytic activities, both peptides completely lost antimicrobial and haemolytic activity. The C-terminus was not chosen as the later modification position because it was discovered that this area lacks the selectivity between antimicrobial activity and haemolytic activity, even anticancer activity, in previous and this study [26,27].

Secondly, two lysines were added onto the N-terminus to increase the antimicrobial activity against Gram-negative bacteria (T1CEh-KK). This strategy has been used in the modification of Temporin B in a previous study [31]. With the molecular dynamics simulation of the interaction between the peptides and the mimic membrane of Gram-positive and Gram-negative bacteria, Temporin B was found to disorder the stability of anionic phosphatidylglycerol (PG). While the modified peptide, Temporin B KKG6A, was found to disorder the stability of zwitterionic phosphatidylethanolamine (PE) which is the maintain component of the plasma membrane of Gram-negative bacteria. In addition, cationic lysine can cluster the anionic lipids efficiently. As the results show, T1CEh-KK increased the anti-Gram-negative bacteria, especially against *E. coli*, as well as maintained the low level of haemolysis.

Thirdly, considering the proline at position 3 is a conserved motif in the Temporin peptide family [32], the aspartic acid was substituted by proline (T1CEh-KKP). It can be expected that the helicity of the N-terminus of the peptide will be broken because of the lack of hydrogen on the α amino group, and the proline cannot achieve a hydrogen bond with another amino acid. However, the increase of flexibility of the N-terminal peptide regions can release the bioactivity potency of this region [33]. The replacement of aspartic acid with proline influenced the helical structure of T1CEh-KKP, which caused a weakening of antimicrobial activity. However, the haemolytic level of T1CEh-KKP was not changed.

What is more, according to a previous study, the indole side chain of tryptophan has a unique interaction with the polar-nonpolar interface, making the peptide anchored in the membrane interface region [34]. Phenylalanine at position 3 and leucine at position 6 of T1CEh-KKP was replaced with tryptophan at a single or both positions (T1CEh-KKPW and T1CEh-KKPWW) in order to increase the antimicrobial activity through applying the special side-chain function and the increase of hydrophobicity. Both peptides showed more potent antimicrobial activity against all tested ESKAPE strains through the use of tryptophan. Except for *E. faecalis*, the minimum inhibitory concentration (MIC) of these two peptides ranged from 4 to 32 μM. The haemolysis rates of these two peptides were lower than 6%.

Finally, the lysine which the side chain protected by the Fmoc group was applied in peptide synthesis to build a peptide with a branched structure. Compared with monomer peptides, branched peptides showed higher bioactivity due to the local concentration of bioactive units being increased [31,32]. In addition, the promotion of steric hindrance made it more resistant to protease degradation, so the pharmacokinetic properties of polypeptides were improved [35]. In this modified strategy, the lysine at position 8 of T1CEh-KKPWW was the branch site and two chains with the same sequence were synthesised and extended from here to N-terminus (T1CEh-KKPWW2). After the achievement of the branched structure, the MIC values of T1CEh-KKPWW2 showed a four-fold decrease against MRSA and *E. faecalis*, as well as a twofold decrease against *P. aeruginosa* and *E. coli*, comparing with that of T1CEh-KKPWW. Even though the haemolytic activity of T1CEh-KKPWW2 experienced an increase comparing with T1CEh-KKPWW (63% and 6% of haemolysis at 256 μM, respectively), the HC_50_ of T1CEh-KKPWW2 was 569.8 μM which was higher than that of Temporin-1CEh.

### 3.4. Secondary Structure Analysis

**Figure 5:**
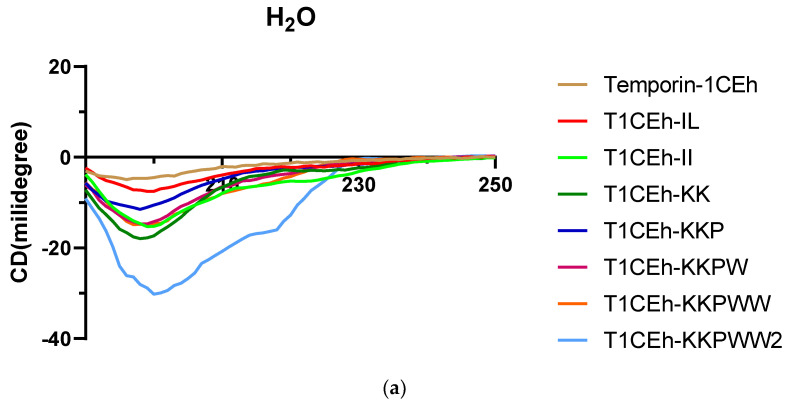

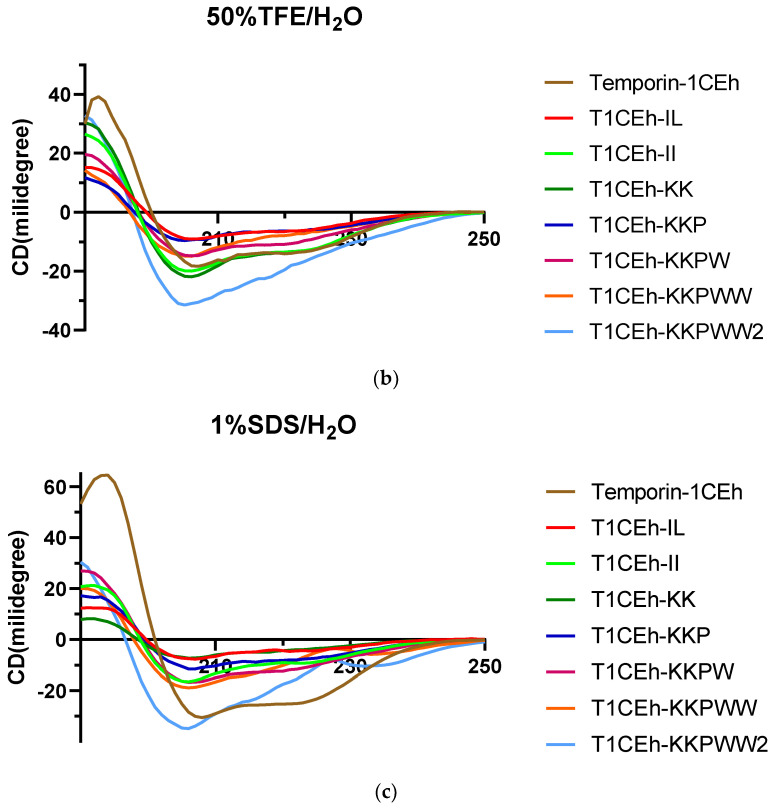
Circular dichroism (CD) spectra of 50 µM of Temporin-1CEh and its analogues in H_2_O (**a**), 50% TFE solution (**b**) and 1% SDS solution (**c**).

**Figure 6:**
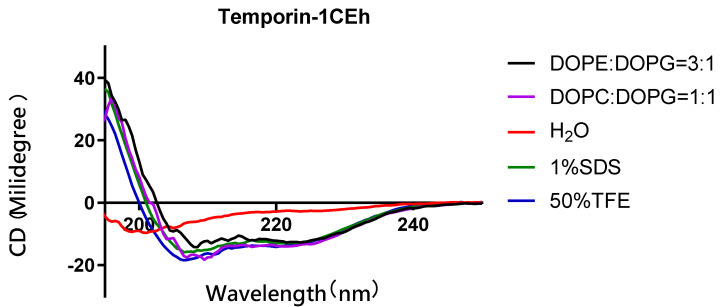
Circular dichroism (CD) spectra of 50 µM of Temporin-1CEh in H2O, 50% TFE solution, 1% SDS solution of *S. aureus* (DOPC:DOPG = 1:1) and *E. coli* (DOPE:DOPG = 3:1)-membrane-mimicking environments.

As the circular dichroism (CD) spectra showed, all peptides formed random coil structures in aqueous environments (Figure 5). However, they formed helical structures in the 50% TFE and 1% SDS environments. Their CD spectra showed two negative peaks at 208 nm and 222 nm of the wavelength, which was the spectra feature of the helical structure. Moreover, Temporin-1CEh formed an alpha-helix with both *S. aureus* (DOPC:DOPG = 1:1) and *E. coli* (DOPE:DOPG = 3:1) liposome constitutions (Figure 6). Temporin-1CEh showed a slight enhancement of helical content in the *S. aureus*-membrane-mimicking model compared to the *E. coli* model as the calculated α-helicity were 65.3% and 45.5%, respectively. The physicochemical characteristics of Temporin-1CEh and its analogues were shown in Table 3.

### 3.5. Membrane Permeabilisation Assays

**Figure 7:**
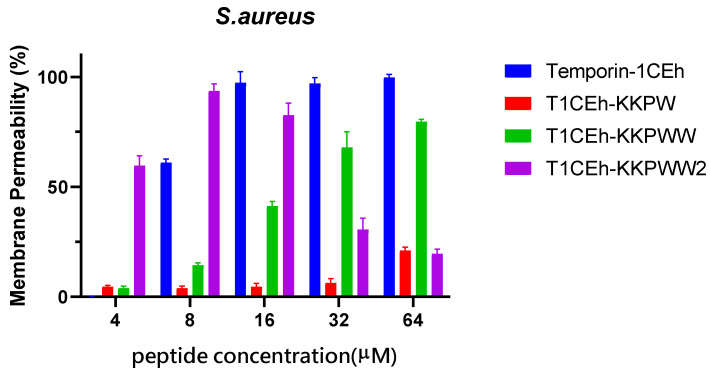

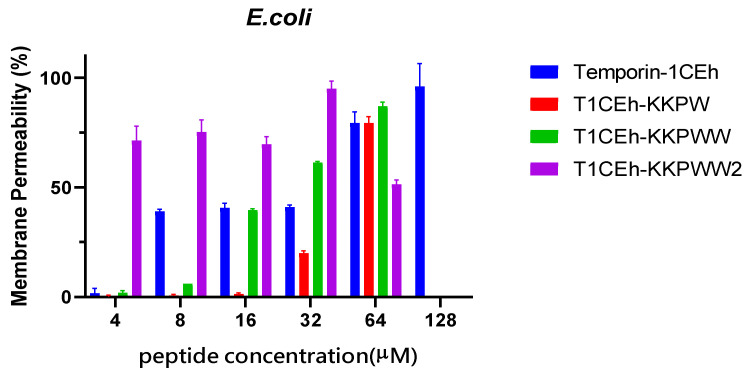
The permeability proportions of Temporin-1CEh, T1CEh-KKPW, T1CEh-KKPWW and T1CEh-KKPWW2 in increasing concentrations on *S. aureus* (ATCC CRM 6538) and *E. coli* (ATCC 8739). The positive and negative controls used 70% isopropanol and 5% tryptic soy Broth (TSB) for *S. aureus* or Lysogeny broth (LB) for *E. coli* in 0.85% NaCl, respectively. Error bars represent the standard error of the mean (SEM) of three replicates.

By measuring the fluorescence intensity when SYTOX GREEN nucleic dye binds to nucleic acid, Temporin-1CEh showed a 100% permeability rate at the MBC (16 μM and 128 μM, respectively) against *S. aureus* and *E. coli* (Figure 7). T1CEh-KKPW and T1CEh-KKPWW showed a significant decrease in the permeability rates against both bacteria at the minimum bactericidal concentration (MBC). With the application of a branched structure, higher permeability rates of T1CEh-KKPWW2, when compared with that of Temporin-1CEh, against *S. aureus* and *E. coli* could be found. The statistical analyses were performed by using the two-way ANOVA test and shown in Tables S1 and S2.

### 3.6. Antibiofilm Assays

**Figure 8:**
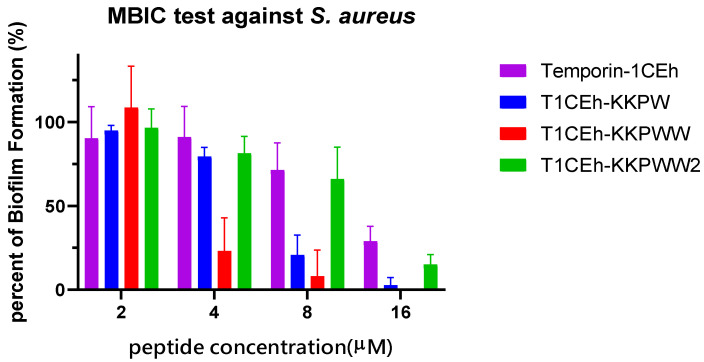

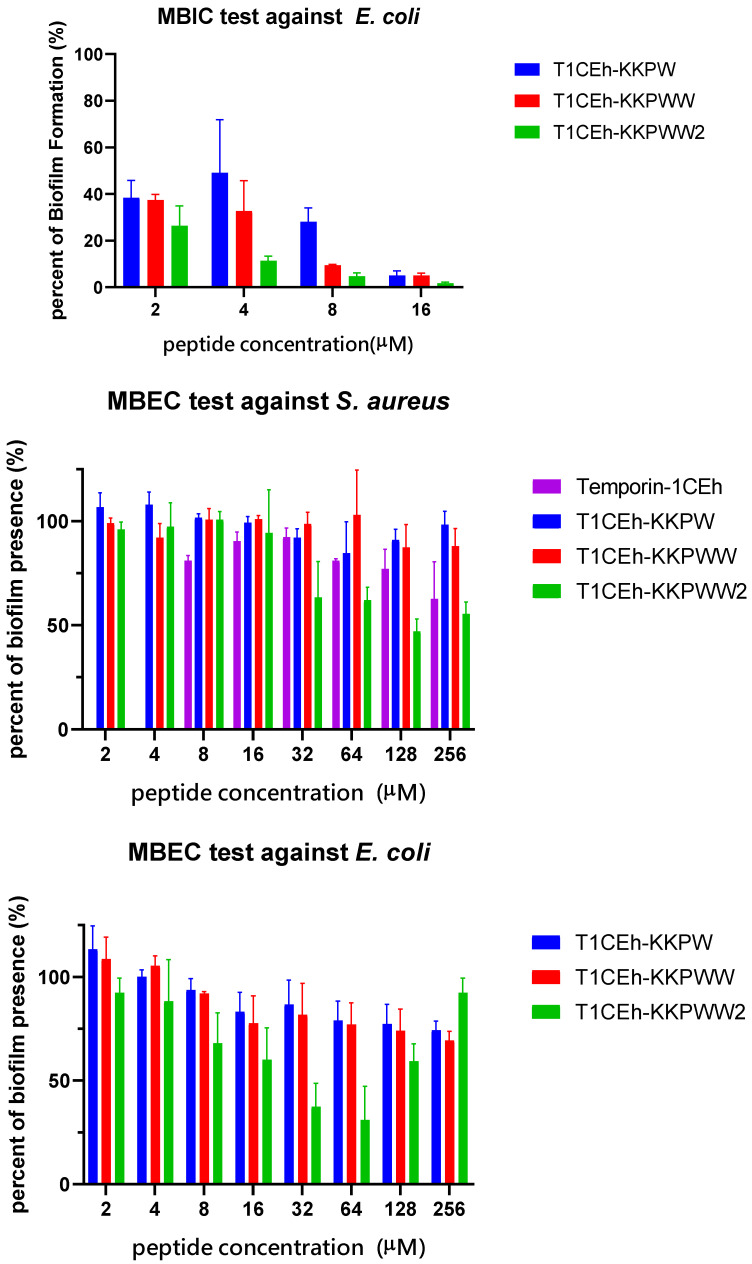
The antibiofilm effects of Temporin-1CEh, T1CEh-KKPW, T1CEh-KKPWW and T1CEh-KKPWW2 against *S. aureus* (ATCC CRM 6538) and *E. coli* (ATCC 8739). Error bars represent the SEM of three replicates.

*S. aureus* (ATCC CRM 6538) and MRSA (NCTC 12493) were employed to study the ability of Temporin-1CEh, T1CEh-KKPW, T1CEh-KKPWW and T1CEh-KKPWW2 to inhibit biofilm formation and eradicate mature biofilm. Four peptides inhibited 90% of biofilm production of *S. aureus* and *E. coli* from 8 to 64 μM (Figure 8). However, all of them at all tested concentrations could not destroy 90% of the mature biofilm. The minimum biofilm inhibitory concentration (MBIC) and minimum biofilm eradication (MBEC) values of the four peptides were shown in Table 4.

### 3.7. Assessing the Impact of Different Environments on Antimicrobial Activity

In order to investigate the effect from acidic and alkalic environments to the ionisation balance of the cationic antimicrobial peptides, pH 6.0 and pH 8.0 environments were used in this assay. In addition, the environment containing serum and different metal cations were used to measure the effect of protein interaction and competitive inhibition, respectively. The antimicrobial activity of Temporin-1CEh experienced a significant decrease in different pH environments or the presence of different salt or FBS environments (Table 5). Compared with the negative effect of pH 8.0, the antimicrobial activity of T1CEh-KKPW, T1CEh-KKPWW and T1CEh-KKPWW2 exhibited a larger decrease at pH 6.0, where MIC values at pH 6.0 were twice or more as much as those at pH 8.0. In addition, unlike T1CEh-KKPW and T1CEh-KKPWW, the antibacterial activity of T1CEh-KKPWW2 was not affected by 10% of FBS or salt conditions.

### 3.8. Assessing the Efficacy of the Peptide against S. aureus and E. coli In Vivo

**Figure 9:**
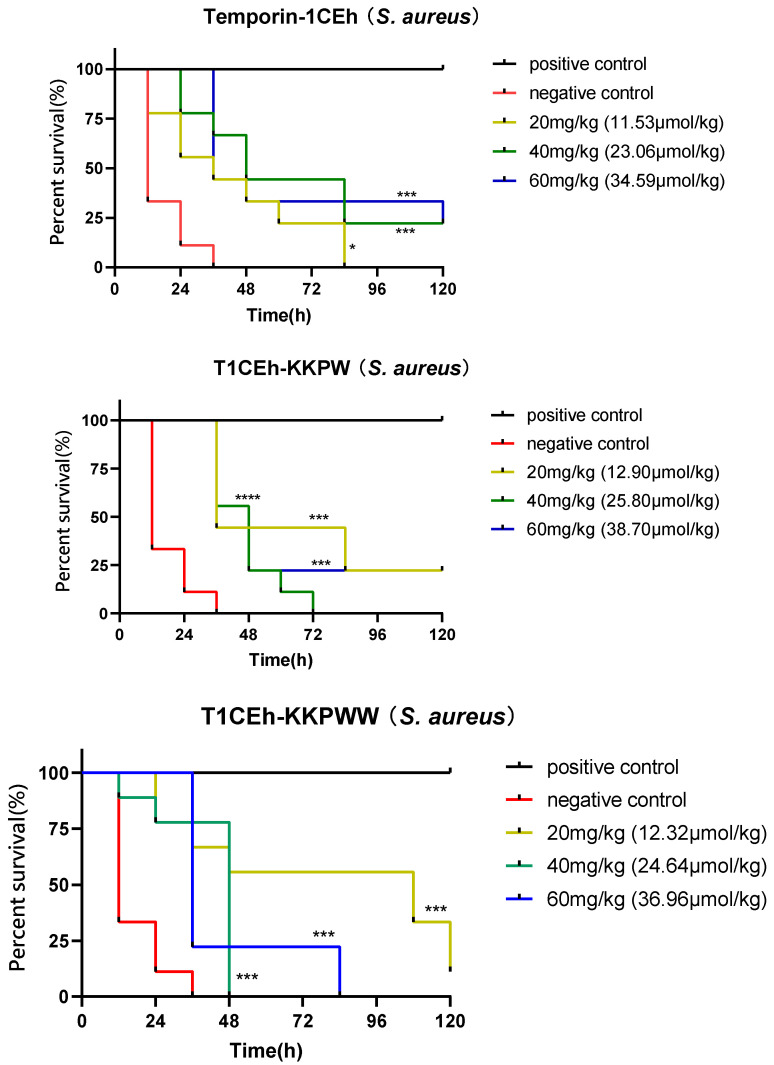

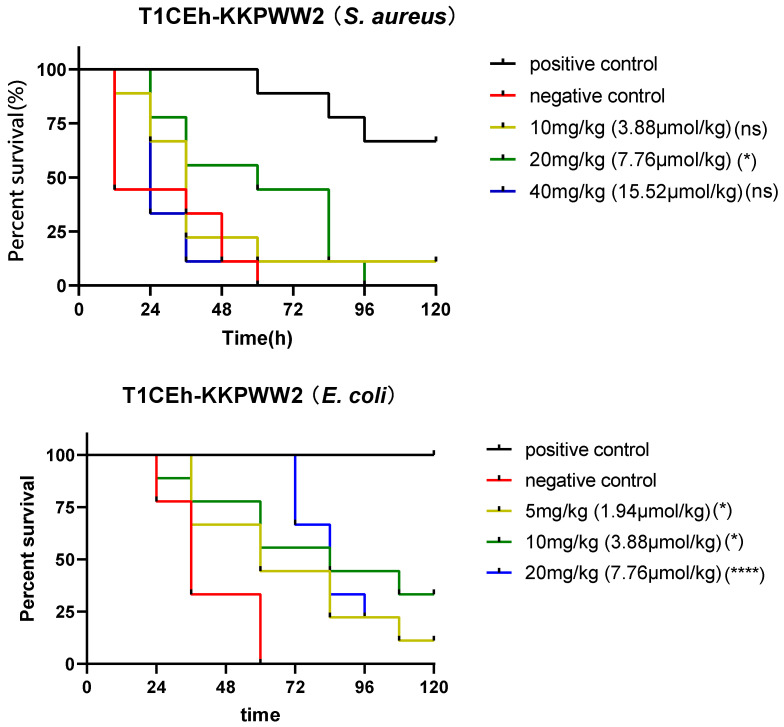
The survival curves of wax moth larvae infected by *S. aureus* and treated by peptide Temporin-1CEh, T1CEh-KKPW, T1CEh-KKPWW and T1CEh-KKPWW2, as well as infected by *E. coli* and treated by peptide T1CEh-KKPWW2. For the treatment of *S. aureus* infection, the positive and negative controls were 50 mg/kg of vancomycin and 0.9% NaCl solution, respectively. For the treatment of *E. coli* infection, the positive and negative controls were 25 mg/kg of ampicillin and 0.9% NaCl solution, respectively. The statistical analyses were performed by using the Mantel–Cox test between the negative group and dose groups, as well as the significant difference was indicated as * (*p* < 0.05), *** (*p* < 0.001) and **** (*p* < 0.0001). The significant levels were indicated in parentheses behind the legend keys of each dose group.

*G. mellonella* larvae were employed as a model to assess the effectiveness of Temporin-1CEh, T1CEh-KKPW and T1CEh-KKPWW against *Staphylococcus* as well as T1CEh-KKPWW2 against *Staphylococcus* and *Escherichia*. Temporin-1CEh, T1CEh-KKPW and T1CEh-KKPWW significantly reduced the mortality of infected larvae. However, there were no significant differences among different peptide concentrations (Figure 9). T1CEh-KKPWW2 significantly reduced the mortality of *S. aureus* infected larvae at a concentration of 20 mg/kg. In addition, T1CEh-KKPWW2 in all tested concentrations reduced the mortality of *E. coli* infected larvae and showed a positive correlation between peptide concentration and survival rate.

## 4. Discussion

It is difficult for bacteria to acquire resistance to antimicrobial peptides because of the direct interaction between bacterial cell membranes and AMPs [37]. Therefore, AMPs as alternative antibiotic candidates have attracted much attention. Temporin, as a large family of antimicrobial peptides, have short sequences which makes their structures and functions easier to be studied [33]. Here, we identified a novel Temporin peptide from the skin secretion of *Rana chensinensis* which consists of 15 amino acids with C-terminal amidation, named Temporin-1CEh. It was noticed that a highly similar peptide, Temporin-1CEa, was found whose amino acid at position 11 is isoleucine.

Similar to Temporin-1CEa, Temporin-1CEh also possesses remarkable antimicrobial activity against Gram-positive bacteria, but is weak at the Gram-negative bacteria. A previous study showed that Temporin induces antimicrobial activity through membrane permeabilisation [38]. Based on this, we assumed that Temporin-1CEh could have different behaviour when interacting with the different cell membranes. As CD spectra showed, Temporin-1CEh possessed a higher proportion of helix in *S. aureus* membranes than those of *E. coli* (Figure 6). The differences in the helical percentage resulted in the differences of antimicrobial potency of Temporin-1CEh against Gram-positive bacteria and Gram-negative bacteria. Another assumption is that the initial interaction of AMPs approaching Gram-positive bacteria and Gram-negative bacteria is different because of the differences of both membrane structures [39]. The electrostatic interactions between cationic AMPs and anionic LPS molecules, as well as the consumption to disrupt both outer and cytoplasmic membranes, weakened the antimicrobial activity of the peptides against Gram-negative bacteria [5,40].

For the bioactivity optimisation of Temporin-1CEh, T1CEh-t and T1CEa-t experienced a significant decrease in calculated helicity in the 50% TFE environment (Table 3) and lost their antimicrobial as well as haemolytic activities. This situation resulted from the removal of hydrophobic amino acids and the loss of the aromatic residues of phenylalanine which can help the peptides anchor to the cell membrane [34]. However, the difference of the amino acids at position 11 of both peptides did not cause the difference in their bioactivities.

As the same situation found in the study of Temporin B KKG6A [31], T1CEh-KK experienced an improvement in antimicrobial activity, especially for the inhibition of the growth of *E. coli*. In addition, the substitution by proline leads to the weakening of the antimicrobial ability of T1CEh-KKP except for against *S. aureus*. However, it was found that α-helical AMPs that possess hinge conformations showed stronger antimicrobial selectivity against Gram-negative bacteria than the linear AMPs [41,42]. The proline at position 14 of melittin was proved to contribute the binding to the membrane as well as the lytic activity [43]. Besides, some peptides that possessed Pro-hinges, such as Buforin II and Anal 3-Pro, penetrated the membranes of bacteria by creating small holes [44,45]. It could be found that the effect by proline to T1CEh-KKP and these peptides with large peptide lengths were different. Buforin II formed a helix–hinge–helix structure, and its helicity percent was 43% in a 50% TFE environment, while that of T1CEh-KKP was 28.8% [44]. The destruction of the helical structure affects the antibacterial activity of T1CEh-KKP (Table 2 and Table 3), which was inconsistent with a previous report of [Pro^3^]-Temporin L which had the same number of amino acids [8]. The high hydrophobicity might result in [Pro^3^]-Temporin L to maintain the antimicrobial activity. In this case, tryptophan was used to increase the hydrophobicity of the peptide and provide the ability to anchor in the membrane. As a result, T1CEh-KKPW and T1CEh-KKPWW significantly showed the comprehensive promotion against Gram-positive and Gram-negative bacteria. At the same time, both peptides showed lower than 10% of a haemolytic rate at 256 μM, maintaining a low haemolytic level with previous modified peptides (Figure 4). In other words, with the insertion of tryptophan, the hydrophobicity was improved without an increase in haemolysis, which implied that hydrophobic interaction with the C-terminus could make more of a contribution to haemolytic activity.

For the further analysis of permeability and antibiofilm ability, the rate of killing of T1CEh-KKPWW against *S. aureus* and *E. coli* was positively related to the peptide concentration from 4 to 64 μM. This membrane-breaking antibacterial mechanism provides the peptide with a good ability to inhibit the biofilm formation showing the MBIC against *S. aureus* and *E. coli* at 8 μM (Table 4). However, after the formation of the biofilm, the biofilm would resist treatment with the T1CEh-KKPWW, which revealed the large gap between its MBIC and MBEC. The mechanism of biofilm resistance to the antimicrobial peptide is not extensively studied [46]. Polysaccharide intercellular adhesin (PIA), a cationic molecule composed of poly-N-acetyl glucosamine, was discovered from extracellular polymeric substances (EPSs) [47]. This molecule provides the electrostatic repulsion for the pathogens to action the treatment from antimicrobial peptides, such as the resistance of *S. epidermidis* and *S. aureus* to dermicidine, LL-37 and human β-defensins [48].

In a previous report, high concentrations of salts, acidic pH and serum could cause a decrease or loss of antimicrobial activity for AMPs [49]. At the same time, considering an in vivo antibacterial activity test, it is worthwhile to consider the antibacterial activity test of peptides under different physiological conditions. The antimicrobial activity of T1CEh-KKPW and T1CEh-KKPWW against ESKAPE pathogens experienced different degrees of influence and the effect of the pH 6 environment was significantly greater than that of pH 8. The acidic environment will inhibit the cationic group on the peptide and then affects the charge–charge interaction in the initial binding step between peptides and bacteria. In addition, T1CEh-KKPWW exhibited a good antimicrobial ability in the environment containing serum which might cause proteolytic digestion [49]. Furthermore, the antimicrobial activity of T1CEh-KKPWW experienced a dramatic decrease in different concentrations of salt, especially against MRSA, *E. faecalis* and *P. aeruginosa*. This phenomenon can be explained that the cations (Na^+^, Mg^2+^ and Fe^3+^) might cause the competitive inhibition to the binding between cationic peptides and bacterial membranes [50].

In order to improve the antibacterial ability of T1CEh-KKPWW in different physiological conditions, a branched structure was used as a modification strategy obtaining peptide T1CEh-KKPWW2. T1CEh-KKPWW2 showed a slight decrease in the antimicrobial activity against ESKAPE pathogens, showing only two- to four-fold weakening of the MIC values. This improvement allowed T1CEh-KKPWW2 to prevent the cation competitive inhibition comparing with T1CEh-KKPWW. It was noticed that the permeability of T1CEh-KKPWW2 at high concentrations showed decreasing trends, which might be due to the binding between peptides and nucleic acids. Previous studies have shown that the peptide LL-37 prevented the degradation of DNA from NETs, purified neutrophil and calf thymus cells derived from bacterial pathogens [51]. In particular, the result directly showed that the fluorescence intensity of the group containing DNA and LL-37 was significantly lower than that only containing DNA.

In addition, the phenomenon of aggregation can be found in T1CEh-KKPWW2 at a high concentration, achieving hydrogel. The aromatic stacking which may cause complicated π−π interactions played a role in the self-assembly in many amyloid-related proteins [52,53]. At the same time, the increase of hydrophobicity which resulted from the accumulation of a hydrophobic amino acid contributed to the aggregation between peptide molecules [54,55]. Too strong an aggregation ability may result in the premature aggregation of the peptide before they are attached to the cell membrane [56,57,58]. This can explain the decrease on the permeability and antibiofilm ability at high concentrations of T1CEh-KKPWW2 (Figure 7 and Figure 8).

The application of branched structure most directly leads to the accumulation of the functional unit to the peptide. Net charges and the hydrophobicity increase of T1CEh-KKPWW2 not only promoted its antimicrobial and antibiofilm activity, but also increased its haemolysis. The same situation could be found in the bioactivity evaluation between branched peptide SB056 and its linear peptide [59]. However, the other two well study branched peptides, G3KL and B2088, were found to have low cell toxicity [45,46,47]. Therefore, peptide sequences play a critical role in haemolysis and cell toxicity rather than the branched structure.

Additionally, the *Galleria mellonella* larvae model demonstrated the potential antimicrobial efficacy of selected peptides in vivo [60]. Temporin-1CEh, T1CEh-KKPW and T1CEh-KKPWW showed good anti-*Staphylococcus* activity in vivo, and all treatment groups exhibited statistical differences compared with the negative control group. However, the reason for no improvement of mortality with increasing doses is not clear. It might be related to the rapid degradation of peptides in vivo. In addition, T1CEh-KKPWW2 showed better anti-*Escherichia* activity in vivo than anti-*Staphylococcus* activity in vivo.

In summary, we reported the study on the characterisation of a novel temporin peptide, the design of the N- and C-terminal domains modified analogues and assessed the influence on their bio-efficacy with in vitro and in vivo models. It revealed that increasing the net charge at the N-terminal domain could improve the selectivity, whilst the hydrophobic interaction with amino acid residues ensures the membrane permeabilisation. The balance of both features is recognised as a key that could be favourable for developing the new therapeutic approaches in the treatment of infectious diseases.

## Figures and Tables

**Table 1:** Peptide sequences of parent peptide Temporin-1CEh and its analogues.

Peptide	Sequence
Temporin-1CEh	FVDLKKIANILNSIF-NH_2_
Temporin-1CEa	FVDLKKIANIINSIF-NH_2_
T1CEh-t	FVDLKKIANIL-NH_2_
T1CEa-t	FVDLKKIANII-NH_2_
T1CEh-KK	KKFVDLKKIANIL-NH_2_
T1CEh-KKP	KKFVPLKKIANIL-NH_2_
T1CEh-KKPW	KKWVPLKKIANIL-NH_2_
T1CEh-KKPWW	KKWVPWKKIANIL-NH_2_
T1CEh-KKPWW2	(KKWVPWK)_2_KIANIL-NH_2_

**Table 2:** The MICs and MBCs of Temporin-1CEh and its analogues as well as Temporin-1CEa against the *S. aureus* (ATCC CRM 6538), MRSA (NCTC 12493), *E. faecalis* (NCTC 12697), *E. coli* (ATCC 8739), *P. aeruginosa* (ATCC 27853) and *K. pneumonia* (ATCC 43816), as well as the MICs of Temporin-1CEa against *S. aureus* and *E. coli* [29].

Bacteria Strains	MIC/MBC (μM)								
	Temporin-1CEh	Temporin-1CEa	T1CEh-t	T1CEa-t	T1CEh-KK	T1CEh-KKP	T1CEh-KKPW	T1CEh-KKPWW	T1CEh-KKPWW2
*S. aureus*	8/16	13.1	>512/>512	>512/>512	256/>512	32/64	8/16	4/4	4/8
MRSA	16/16	-	>512/>512	>512/>512	>512/>512	256/256	16/64	32/256	8/16
*E. faecalis*	32/32	-	>512/>512	>512/>512	64/>512	>512/>512	>512/>512	128/128	32/64
*K. pneumoniae*	256/256	-	>512/>512	>512/>512	128/128	>512/>512	16/16	4/4	4/8
*P. aeruginosa*	128/256	-	>512/>512	>512/>512	256/256	512/>512	32/64	16/16	8/16
*E. coli*	128/128	>100	>512/>512	>512/>512	8/16	128/128	8/16	4/4	2/2

**Table 3:** Physicochemical characteristics of Temporin-1CEh and its analogues. Each amino acid chain is assigned a value, positive or negative, according to its hydrophobicity. These values are used to weigh vectors for each residue as they are displayed around the helix. The summation of the vectors is the hydrophobic moment. The sum of the hydrophobicity values divided by the number of the residues. This value is hydrophobicity. Alpha-helicity was calculated from the data of CD value for respective peptides in 50/50 TFE/H_2_O (*v*/*v*) environment by the Bestsel website [36]. It has not found published data of α-helicity value of Temporin-1CEa.

Peptide	Hydrophobicity (H)	Hydrophobic Moment (μH)	Net Charge (z)	α-Helicity (%)
Temporin-1CEh	0.661	0.518	1	47.8
Temporin-1CEa	0.668	0.524	1	-
T1CEh-t	0.634	0.481	1	33
T1CEa-t	0.643	0.490	1	40.2
T1CEh-KK	0.384	0.385	3	46.2
T1CEh-KKP	0.498	0.394	4	28.8
T1CEh-KKPW	0.534	0.400	4	40.4
T1CEh-KKPWW	0.576	0.440	4	30.2

**Table 4:** MBIC and MBEC of Temporin-1CEh, T1CEh-KKPW, T1CEh-KKPWW and T1CEh-KKPWW2 against *S. aureus* (ATCC CRM 6538) and *E. coli* (ATCC 8739). The peptide concentrations which inhibited more than 90% of biofilm formation or eradicated more than 90% of mature biofilm, are defined as the MBIC and MBEC, respectively.

Bacteria Strains	MBIC/MBEC (µM)			
	Temporin-1CEh	T1CEh-KKPW	T1CEh-KKPWW	T1CEh-KKPWW2
*S. aureus*	64/>256	16/>256	8/>256	32/>256
*E. coli*	-	16/>256	8/>256	8/>256

**Table 5:** MIC values of Temporin-1CEh, T1CEh-KKPW, T1CEh-KKPWW and T1CEh-KKPWW2 against six pathogens at pH 6.0, 7.0 and 8.0 MHB medium environment, as well as the MHB medium respectively contain 10% of FBS, 150 mM of NaCl, 1 mM of MgCl_2_ and 4 µM of FeCl_3_. *S. aureus* (ATCC CRM 6538), MRSA (NCTC 12493), *E. faecalis* (NCTC 12697), *E. coli* (ATCC 8739), *P. aeruginosa* (ATCC 27853) and *K. pneumoniae* (ATCC 43816) were used in this assay.

Peptide	Bacteria	MIC (μM)						
		pH 6.0	pH 7.0	pH8.0	MHB + 10% FBS	MHB + 150 mM NaCl	MHB + 1 mM MgCl_2_	MHB + 4 µM FeCl_3_
Temporin-1CEh	*S. aureus*	128	8	128	128	128	128	128
	MRSA	256	16	256	256	256	128	128
	*E. faecalis*	>512	32	>512	>512	>512	128	128
	*K. pneumoniae*	>512	256	>512	>512	>512	>512	>512
	*P. aeruginosa*	>512	128	>512	>512	>512	>512	>512
	*E. coli*	>512	128	>512	>512	>512	>512	>512
T1CEh-KKPW	*S. aureus*	>512	8	32	>512	>512	256	>512
	MRSA	>512	16	128	>512	>512	>512	>512
	*E. faecalis*	>512	>512	256	512	>512	>512	>512
	*K. pneumoniae*	256	16	128	256	>512	16	16
	*P. aeruginosa*	64	32	32	64	>512	>512	>512
	*E. coli*	16	8	8	8	128	32	64
T1CEh-KKPWW	*S. aureus*	32	4	4	128	16	32	64
	MRSA	256	32	32	64	>512	>512	512
	*E. faecalis*	>512	128	64	128	>512	512	512
	*K. pneumoniae*	32	4	8	64	256	16	8
	*P. aeruginosa*	16	16	4	32	>512	>512	>512
	*E. coli*	4	4	4	4	8	8	8
T1CEh-KKPWW2	*S. aureus*	8	4	2	4	16	8	4
	MRSA	16	8	2	8	8	8	4
	*E. faecalis*	128	32	64	64	128	64	64
	*K. pneumoniae*	8	4	4	16	16	8	8
	*P. aeruginosa*	32	8	8	32	32	16	16
	*E. coli*	2	2	2	4	2	4	2

## Data Availability

The mature peptide identified from the skin secretion was named Temporin-1CEh, and the nucleotide sequence of cDNA has been deposited in the Genbank database under an accession number: OM240644.

## Supplementary Materials

The following supporting information can be downloaded at: https://www.mdpi.com/article/10.3390/pharmaceutics14030604/s1, Table S1: Two-way ANOVA analysis of permeability of Temporin-1CEh and its analogues against *S. aureus*. The significance was presented by the symbol ns (non-significant difference), * (*p* < 0.05), ** (*p* < 0.01), *** (*p* < 0.001) and **** (*p* < 0.0001); Table S2: Two-way ANOVA analysis of permeability of Temporin-1CEh and its analogues against *E. coli*. The significance was presented by the symbol ns (non-significant difference), * (*p* < 0.05), ** (*p* < 0.01), *** (*p* < 0.001) and **** (*p* < 0.0001).
