# Supplementary material for: In Vitro & In Vivo Studies on Identifying and Designing Temporin-1CEh from the Skin Secretion of Rana chensinensis as the Optimised Antibacterial Prototype Drug
Source: Pharmaceutics. 2022 Mar 10;14(3):604. doi: 10.3390/pharmaceutics14030604 (PMC8949600; doi:10.3390/pharmaceutics14030604)
Supplement: Supplementary file 1 [file pharmaceutics-14-00604-s001.zip › pharmaceutics-1589150-supplementary.pdf]

# Supplementary Materials

# *In Vitro* & *In Vivo* studies on Identifying and Designing Temporin-1CEh from the Skin Secretion of *Rana chensinensis* as the Optimized Antibacterial Prototype Drug

**Table S1**. Two-way ANOVA analysis of permeability of Temporin-1CEh and its analogues against *S. aureus*. The significance was presented by the symbol ns (non-significant difference), ** (P<0.01) and **** (P<0.0001).

| Tukey's multiple comparisons test | | | | | |
|---|---|---|---|---|---|
| Peptide concentration (μM) | Peptide name | Mean Diff. | 95.00% CI of diff. | Summary | Adjusted P Value |
| 4 | Temporin-1CEh vs. T1CEh-KKPW | -5 | -11.90 to 1.898 | ns | 0.2269 |
| | Temporin-1CEh vs. T1CEh-KKPWW | -4.333 | -11.23 to 2.564 | ns | 0.3454 |
| | Temporin-1CEh vs. T1CEh-KKPWW2 | -60 | -66.90 to -53.10 | **** | <0.0001 |
| | T1CEh-KKPW vs. T1CEh-KKPWW | 0.6667 | -6.231 to 7.564 | ns | 0.9938 |
| | T1CEh-KKPW vs. T1CEh-KKPWW2 | -55 | -61.90 to -48.10 | **** | <0.0001 |
| | T1CEh-KKPWW vs. T1CEh-KKPWW2 | -55.67 | -62.56 to -48.77 | **** | <0.0001 |
| 8 | Temporin-1CEh vs. T1CEh-KKPW | 57 | 50.10 to 63.90 | **** | <0.0001 |
| | Temporin-1CEh vs. T1CEh-KKPWW | 46.67 | 39.77 to 53.56 | **** | <0.0001 |
| | Temporin-1CEh vs. T1CEh-KKPWW2 | -32.67 | -39.56 to -25.77 | **** | <0.0001 |
| | T1CEh-KKPW vs. T1CEh-KKPWW | -10.33 | -17.23 to -3.436 | ** | 0.0014 |
| | T1CEh-KKPW vs. T1CEh-KKPWW2 | -89.67 | -96.56 to -82.77 | **** | <0.0001 |
| | T1CEh-KKPWW vs. T1CEh-KKPWW2 | -79.33 | -86.23 to -72.44 | **** | <0.0001 |
| 16 | Temporin-1CEh vs. T1CEh-KKPW | 92.67 | 85.77 to 99.56 | **** | <0.0001 |
| | Temporin-1CEh vs. T1CEh-KKPWW | 56 | 49.10 to 62.90 | **** | <0.0001 |
| | Temporin-1CEh vs. T1CEh-KKPWW2 | 14.67 | 7.769 to 21.56 | **** | <0.0001 |
| | T1CEh-KKPW vs. T1CEh-KKPWW | -36.67 | -43.56 to -29.77 | **** | <0.0001 |

| | | | | | |
|---|---|---|---|---|---|
| | T1CEh-KKPW vs. T1CEh-KKPWW2 | -78 | -84.90 to -71.10 | **** | <0.0001 |
| | T1CEh-KKPWW vs. T1CEh-KKPWW2 | -41.33 | -48.23 to -34.44 | **** | <0.0001 |
| 32 | Temporin-1CEh vs. T1CEh-KKPW | 90.67 | 83.77 to 97.56 | **** | <0.0001 |
| | Temporin-1CEh vs. T1CEh-KKPWW | 29 | 22.10 to 35.90 | **** | <0.0001 |
| | Temporin-1CEh vs. T1CEh-KKPWW2 | 66.33 | 59.44 to 73.23 | **** | <0.0001 |
| | T1CEh-KKPW vs. T1CEh-KKPWW | -61.67 | -68.56 to -54.77 | **** | <0.0001 |
| | T1CEh-KKPW vs. T1CEh-KKPWW2 | -24.33 | -31.23 to -17.44 | **** | <0.0001 |
| | T1CEh-KKPWW vs. T1CEh-KKPWW2 | 37.33 | 30.44 to 44.23 | **** | <0.0001 |
| 64 | Temporin-1CEh vs. T1CEh-KKPW | 78.67 | 71.77 to 85.56 | **** | <0.0001 |
| | Temporin-1CEh vs. T1CEh-KKPWW | 20 | 13.10 to 26.90 | **** | <0.0001 |
| | Temporin-1CEh vs. T1CEh-KKPWW2 | 80 | 73.10 to 86.90 | **** | <0.0001 |
| | T1CEh-KKPW vs. T1CEh-KKPWW | -58.67 | -65.56 to -51.77 | **** | <0.0001 |
| | T1CEh-KKPW vs. T1CEh-KKPWW2 | 1.333 | -5.564 to 8.231 | ns | 0.9543 |
| | T1CEh-KKPWW vs. T1CEh-KKPWW2 | 60 | 53.10 to 66.90 | **** | <0.0001 |

**Table S2**. Two-way ANOVA analysis of permeability of Temporin-1CEh and its analogues against *E. coli*. The significance was presented by the symbol ns (non-significant difference), ** ($P<0.01$) and **** ($P<0.0001$).

| Tukey's multiple comparisons test | | | | | |
|---|---|---|---|---|---|
| Peptide concentration (μM) | Peptide name | Mean Diff. | 95.00% CI of diff. | Summary | Adjusted P Value |
| 4 | Temporin-1CEh vs. T1CEh-KKPW | 1.333 | -4.825 to 7.491 | ns | 0.9374 |
| | Temporin-1CEh vs. T1CEh-KKPWW | -0.3333 | -6.491 to 5.825 | ns | 0.9989 |
| | Temporin-1CEh vs. T1CEh-KKPWW2 | -69.67 | -75.82 to -63.51 | **** | <0.0001 |
| | T1CEh-KKPW vs. T1CEh-KKPWW | -1.667 | -7.825 to 4.491 | ns | 0.8863 |
| | T1CEh-KKPW vs. T1CEh-KKPWW2 | -71 | -77.16 to -64.84 | **** | <0.0001 |
| | T1CEh-KKPWW vs. T1CEh-KKPWW2 | -69.33 | -75.49 to -63.18 | **** | <0.0001 |
| 8 | Temporin-1CEh vs. T1CEh-KKPW | 38.33 | 32.18 to 44.49 | **** | <0.0001 |

| | | | | | |
|---|---|---|---|---|---|
| | Temporin-1CEh vs. T1CEh-KKPWW | 33 | 26.84 to 39.16 | **** | <0.0001 |
| | Temporin-1CEh vs. T1CEh-KKPWW2 | -36.33 | -42.49 to -30.18 | **** | <0.0001 |
| | T1CEh-KKPW vs. T1CEh-KKPWW | -5.333 | -11.49 to 0.8245 | ns | 0.1101 |
| | T1CEh-KKPW vs. T1CEh-KKPWW2 | -74.67 | -80.82 to -68.51 | **** | <0.0001 |
| | T1CEh-KKPWW vs. T1CEh-KKPWW2 | -69.33 | -75.49 to -63.18 | **** | <0.0001 |
| 16 | Temporin-1CEh vs. T1CEh-KKPW | 39.33 | 33.18 to 45.49 | **** | <0.0001 |
| | Temporin-1CEh vs. T1CEh-KKPWW | 1 | -5.158 to 7.158 | ns | 0.972 |
| | Temporin-1CEh vs. T1CEh-KKPWW2 | -29 | -35.16 to -22.84 | **** | <0.0001 |
| | T1CEh-KKPW vs. T1CEh-KKPWW | -38.33 | -44.49 to -32.18 | **** | <0.0001 |
| | T1CEh-KKPW vs. T1CEh-KKPWW2 | -68.33 | -74.49 to -62.18 | **** | <0.0001 |
| | T1CEh-KKPWW vs. T1CEh-KKPWW2 | -30 | -36.16 to -23.84 | **** | <0.0001 |
| 32 | Temporin-1CEh vs. T1CEh-KKPW | 21 | 14.84 to 27.16 | **** | <0.0001 |
| | Temporin-1CEh vs. T1CEh-KKPWW | -20.33 | -26.49 to -14.18 | **** | <0.0001 |
| | Temporin-1CEh vs. T1CEh-KKPWW2 | -54 | -60.16 to -47.84 | **** | <0.0001 |
| | T1CEh-KKPW vs. T1CEh-KKPWW | -41.33 | -47.49 to -35.18 | **** | <0.0001 |
| | T1CEh-KKPW vs. T1CEh-KKPWW2 | -75 | -81.16 to -68.84 | **** | <0.0001 |
| | T1CEh-KKPWW vs. T1CEh-KKPWW2 | -33.67 | -39.82 to -27.51 | **** | <0.0001 |
| 64 | Temporin-1CEh vs. T1CEh-KKPW | 0 | -6.158 to 6.158 | ns | >0.9999 |
| | Temporin-1CEh vs. T1CEh-KKPWW | -7.667 | -13.82 to -1.509 | ** | 0.0095 |
| | Temporin-1CEh vs. T1CEh-KKPWW2 | 28 | 21.84 to 34.16 | **** | <0.0001 |
| | T1CEh-KKPW vs. T1CEh-KKPWW | -7.667 | -13.82 to -1.509 | ** | 0.0095 |
| | T1CEh-KKPW vs. T1CEh-KKPWW2 | 28 | 21.84 to 34.16 | **** | <0.0001 |
| | T1CEh-KKPWW vs. T1CEh-KKPWW2 | 35.67 | 29.51 to 41.82 | **** | <0.0001 |
